# Scarlet Fever in a Family of Five Children

**Published:** 1889-12-14

**Authors:** 


### THE PRACTITIONER'S MIRROR OF CASES.

[Cases for publication in the Mirror should reach the Editor, The Lodge, Porchester-square, W., not later than Friday morning in each week.]

### SCARLET FEVER IN A FAMILY OF FIVE CHILDREN. EACH CASE ABORTED IN SEVEN DAYS BY THE BINIODIDE OF MERCURY.

Robert Kay, aged nine, began on September 30, with sore throat and fever. Summoned on the following day, in the evening, I found him suffering from scarlatina anginosa, and the rash well marked. I gave the following mixture: sol. hyd. bichlor. ʒvi, pot. iod. gr. xv, sp. am. co. ʒi, syrup. ℥ss, aq. ad. ℥vi. ℥ss secundis horis. Throat to be sprayed every four hours with 1 in 2,000 biniodide in sodic iodide. Oct. 2. Rash fully out; restless, sleepless, and very fever ish. 6th. The following note made: "The rash became less and less marked each day, until now there is no trace of it. Healthy granulation of the ulcerated tonsils. Pharyngeal glandular swelling externally, decreasing. Bowels moved once each day. Temperature normal." The rest of the family occupied the same room. 9th,

Medicine changed to iron and chlorate of potash with biniodide in $\frac{1}{16}$th grain powders three times a day. Sarah Ann, aged three, began with scarlatinal throat, but no rash, furred tongue, vomiting, and feverishness, and was treated as for quinsy. 10th. Stephen, aged five, began with high fever (102deg.) and rash all over the body. Ordered the following: sol. hyd. bichlor. ℥ss, pot. iod. gr. viii., sp. am. co. ʒss., syrup ʒiii., aq. ad. ℥iss. ʒi secundis horis. 11th. Stephen passed a bad night. Objects to the spray. Temperature 102·7deg. Throat much inflamed, and covered with exudation. The application of a 1 in 500 solution of biniodide by laryngeal wire-handled camel's-hair brush to the throat ordered three times a day, the father undertaking to see to it. Sarah Ann's case now one of well-marked fever, with rash, and treated similarly. 13th. John, aged ten, began with high fever, sore throat, and rash. Had same mixture as Robert. Tonsils and pharynx much inflamed, and covered with exuded lymph. 14th. All doing well except John, who began with purging in the night. The mixture was at once stopped, and the liquor ferri perchlor. fort. in 2½ minim doses ordered, in glycerine and water, every 1½ or two hours, and the biniodide in $\frac{1}{16}$th grain powders three times a day. 15th. John's diarrhœa was rapidly cured by the iron. Each case had throat-painting with 1-500 every four hours. Stephen's skin quite free from rash, temperature 99deg.; sleeps and eats well. 16th. Stephen's temperature 99deg. John improving. Sarah Ann to get up. On the 11th, Maud, aged two years, had inflamed throat and took five or six doses of the biniodide mixture, but no rash ever showed itself, so little notice was taken of the child. 20th. John's temperature normal. 24th. Robert, having passed the twenty-second day without kidney symptoms, may be considered free from the danger of any sequelæ. The slightest scurfy desquamation noticeable, and that chiefly affecting the fingers and toes. 28th. Stephen, after severe earache and swelling under the ear, had a discharge of matter from the left ear. On the 30th I was called in and prescribed the 1 in 2,000 lotion to be dropped in, and the mixture with ammonia citrate of iron to be taken three times a day for the pain remaining. Maud, who had no throat-painting, was now suffering from a large abscess in the neck. Poultices were applied and it was incised on November 6th, a large quantity of pus being evacuated. It healed in ten days with carbolic ointment. Remarks. All four cases had the temperature reduced to the normal in six days, and were convalescent on the seventh. None had dropsy. In one, not at first "throat-painted," the disease caused otitis by extension along the Eustachian tube. The only case of cervical abscess was one in which there had been no local application made. In one, John, urgent purging came on within twenty-four hours, and the throat lesion was noticed to be pharyngeal rather than tonsillar. This was immediately checked by iron frequently given. As regards infection, I have since found that by painting the throat with the 1 in 500 solution every four hours the disease is prevented from spreading. Desquamation in all was merely scurfy, and chiefly affected the finger and toes.

C. R. Illingworth, M.D., M.R.C.S.

